# Degeneracy

**Published:** 1905-04-15

**Authors:** E. S. Talbot


### DEGENERACY.

BY E. S. TALBOT, M.D., D.D.S.

It is one of the hardest things in the world to make a person who has not given much thought to this subject understand exactly what it meant by degeneracy. A person may have a degenerate brain; he may have an arrested development of brain. What is generally termed a degenerate may be a criminal, a thief, a liar, and so on. But we have degenerate structures. Man cannot evolute, he cannot develop, without losing some structures and having others develop; hence the vermiform appendix is an organ that is passing; the pituitary gland, the small ribs, the little toes, the little muscles of the ears are not being used, and therefore nature is getting rid of them for higher mental development. They are degenerative structures. It is the brain that is developing in the human race, or, as the gentleman said here, certain nations have become arrested and they are dying out. I have not time to explain it, but he is quite right about that—that a nation will go on to a certain period and if there is no new blood infused into it from other nations that nation will soon die out; if it takes a thousand years, or perhaps two thousand years, nevertheless it will do it. But here in America we have an advantage over any other nation of the globe; individuals are pouring in from all over the world, and we are intermarrying and furnishing new blood, and for that reason the brains are developing in this country, which up to a certain time, as soon as immigration ceases, and we intermarry among our own relatives, as noticed in the nobility of Europe, will then bring about mental degeneration.

But, on the other hand, the face—the teeth are not being used, the face is not being used, the eyes are not being used, the nose is not used, and that is the reason why the face is degenerating for the benefit of the brain. We usually do most of our work by machinery now; we do very little of it by hand. We do not knaw upon trees with our teeth as the lower animals do, for instance; but the brain is developed, our work is being done by machinery, and the physical part of the human body is degenerating. We are going on in a higher evolution, and that is the reason why degeneration of the face is going right on; that is why we have irregularities of the teeth; we are going right on all the time. We are not stopping decay of the teeth in the race; we are only stopping it in individuals; we are not stopping it as a nation or as a family. Every generation that comes into this world will have more decay of the teeth than the generation before. The same is true with the irregularities of the teeth. They are going to become more prominent as we ascend the scale of evolution, because of the recession of the jaws, and because we do not use them.

This is a proper conception of the whole subject. We have to contend with decay of the teeth, with irregularities of the teeth, with interstitial gingivitis; and all of these conditions are based on this law of economy of growth. The structure of an organ is lost for the benefit of the organism as a whole. Now, then, where a parent or a child has a nervous condition we get this arrested development. It may be due to going to parties too much of evenings and sitting up nights and sleeping in the daytime; it may be due to overwork; it may be due to disease that is inherited. If, however, the mother be a strong, healthy woman she may be able to ward off these conditions that we are called upon to treat. If, on the other hand, her nervous system is unstrung and the child does not get proper nourishment, owiug to an unstable nervous system, then we have all these condnions to which I have called your attention.—*Cosmos.*

